# Insuficiência Pulmonar na Insuficiência Cardíaca: Causalidade ou Marcador de Gravidade da Doença?

**DOI:** 10.36660/abc.20260265

**Published:** 2026-07-30

**Authors:** Kayihan Karaman, Metin Karayakali

**Affiliations:** 1 Tokat Gaziosmanpaşa University Tokat Turquia Tokat Gaziosmanpaşa University, Tokat – Turquia

**Keywords:** Insuficiência Cardíaca, Teste de Caminhada, Peptídeos Natriuréticos

Prezado Editor,

Lemos com grande interesse o artigo de Aydin et al.^1^ que investiga a associação entre a gravidade da regurgitação da valva pulmonar (RP) e os níveis de pró-peptídeo natriurético tipo B N-terminal (pró-BNP), a distância percorrida no teste de caminhada de seis minutos (TC6M) e as complicações em 490 pacientes com insuficiência cardíaca (IC). Parabenizamos os autores por abordarem essa patologia valvar pouco explorada; contudo, diversas questões metodológicas e de interpretação exigem uma análise cuidadosa antes que as conclusões possam ser plenamente aceitas.

## 1. Desequilíbrio severo entre grupos e poder estatístico

A notável disparidade no tamanho dos grupos — 272 (sem RP) e 162 (RP leve) versus apenas 20 (RP moderada) e 36 (RP grave) — levanta sérias preocupações sobre o poder estatístico dos modelos multivariados.² Em um modelo multivariado com cinco preditores (gravidade da RP, idade, FEVE, PSAP e hemoglobina), a confiabilidade dos coeficientes derivados para os subgrupos menores pode ser comprometida pela falta de poder estatístico. Esse desequilíbrio levanta preocupações adicionais quanto à precisão e estabilidade dos coeficientes de regressão relatados (β = 0,48 para pró-BNP e β = −0,39 para o teste de caminhada de 6 minutos (TC6M)). Instamos os autores a relatarem intervalos de confiança de 95% para todos os coeficientes beta e a considerarem análises de sensibilidade baseadas em bootstrap para avaliar a robustez dos resultados.

## 2. Confiabilidade da classificação ecocardiográfica

Os autores reconhecem que não foi realizada uma avaliação sistemática da reprodutibilidade interobservador. Sem a estatística kappa, a confiabilidade da classificação semiquantitativa da regurgitação pulmonar não pode ser verificada, especialmente considerando que a largura da vena contracta foi o único parâmetro quantitativo disponível. Além disso, a ausência de outros parâmetros quantitativos — como o volume ou a fração regurgitante — limita a validade de um sistema de classificação de quatro graus quando baseado unicamente na largura da vena contracta.^3^

## 3. Viés de seleção em dados do teste de caminhada de 6 minutos

O TC6M foi realizado apenas "quando disponível", criando um risco substancial de viés de seleção. Pacientes com a limitação funcional mais grave — que têm maior probabilidade de estar nos grupos de RP Moderada e Grave — podem ter sido sistematicamente excluídos do teste, potencialmente subestimando o comprometimento funcional nesses grupos.^4^ Os autores devem relatar as taxas de conclusão por grupo de gravidade da RP e as comparações basais entre pacientes testados e não testados.

## 4. Fatores de confusão não mensurados e linguagem causal

O modelo multivariado omite importantes fatores de confusão — incluindo índice de massa corporal, etiologia da IC, carga de fibrilação atrial e terapias contemporâneas para IC (inibidores de SGLT2, sacubitril/valsartana) — que influenciam independentemente tanto o pró-BNP quanto o TC6M. Além disso, a discussão emprega uma linguagem causal e mecanicista que um estudo transversal retrospectivo não pode sustentar. É igualmente plausível que a IC avançada leve à hipertensão pulmonar secundária, dilatação do anel mitral e, consequentemente, maior regurgitação pulmonar — a via causal inversa. Recomendamos substituir consistentemente a linguagem causal por uma linguagem associativa ao longo de todo o manuscrito. As implicações terapêuticas devem ser claramente apresentadas como geradoras de hipóteses, e não como orientadoras da prática clínica.

Em resumo, Aydin et al.^1^ abordam uma questão clinicamente relevante e apresentam achados de interesse para a comunidade de IC. A resolução das questões estatísticas, metodológicas e interpretativas descritas acima aumentaria substancialmente a validade das conclusões do manuscrito. Aguardamos com expectativa estudos prospectivos que estabeleçam de forma mais definitiva o papel prognóstico da gravidade da regurgitação pulmonar em populações com IC.
