# Supplementary material for: Insuficiência Pulmonar na Insuficiência Cardíaca: Causalidade ou Marcador de Gravidade da Doença?
Source: Arq Bras Cardiol. 2026 Jul 30;123(7):e20260265. [Article in Portuguese] doi: 10.36660/abc.20260265 (PMC13668446; doi:10.36660/abc.20260265)
Supplement: Translation

# Translation: Pulmonary Regurgitation in Heart Failure: Causality or a Marker of Disease Severity?

**DOI:** 10.36660/abc.20260265i

**Authors:** Kayihan Karaman, Metin Karayakali

**Affiliations:** 1 Tokat Gaziosmanpaşa University Tokat Türkiye Tokat Gaziosmanpaşa University, Tokat – Türkiye

**Keywords:** Heart Failure, Walk Test, Natriuretic Peptides

**Dear Editor,**

We read with great interest the article by Aydin et al.^1^ which investigates the association between pulmonary valve regurgitation (PR) severity and levels of N-terminal pro-B-type natriuretic peptide (pro-BNP), six-minute walk test (6MWT) distance, and complications in 490 heart failure (HF) patients. We commend the authors for addressing this underexplored valvular pathology; however, several methodological and interpretive concerns warrant careful consideration before the conclusions can be fully accepted.

## 1. Severe Group Imbalance and Statistical Power

The striking disparity in group sizes—272 (No PR) and 162 (Mild PR) versus only 20 (Moderate PR) and 36 (Severe PR)—raises serious concerns about the statistical power of the multivariate models.^2^ In a multivariate model featuring five predictors (PR severity, age, LVEF, PASP, and hemoglobin), the reliability of the derived coefficients for the smaller subgroups may be compromised by the lack of statistical power. This imbalance raises additional concerns regarding the precision and stability of the reported regression coefficients (β=0.48 for pro-BNP and β=−0.39 for 6MWT). We urge the authors to report 95% confidence intervals for all beta coefficients and to consider bootstrap-based sensitivity analyses to assess the robustness.

## 2. Echocardiographic Grading Reliability

The authors acknowledge that a systematic interobserver reproducibility assessment was not performed. Without kappa statistics, the reliability of the semi-quantitative PR grading cannot be verified, particularly given that vena contracta width was the only quantitative parameter available. Furthermore, the lack of other quantitative parameters—such as regurgitant volume or fraction—limits the validity of a four-grade classification system when based solely on vena contracta width.^3^

## 3. Selection Bias in 6MWT Data

The 6MWT was performed only "when available," creating a substantial risk of ascertainment bias. Patients with the most severe functional limitation—who are more likely to be in the Moderate and Severe PR groups—may have been systematically excluded from testing, potentially underestimating functional impairment in these groups.^4^ The authors should report completion rates per PR severity group and baseline comparisons between tested and non-tested patients.

## 4. Unmeasured Confounders and Causal Language

The multivariate model omits key confounders—including body mass index, HF etiology, atrial fibrillation burden, and contemporary HF therapies (SGLT2 inhibitors, sacubitril/valsartan)—that independently influence both pro-BNP and 6MWT. Moreover, the discussion employs causal and mechanistic language that a cross-sectional retrospective design cannot support. It is equally plausible that advanced HF leads to secondary pulmonary hypertension, annular dilation, and consequently more PR—the reverse causal pathway. We recommend consistently replacing causal wording with associative language throughout the manuscript. Therapeutic implications should be clearly framed as hypothesis-generating rather than practice-guiding.

In summary, Aydin et al.^1^ address a clinically relevant question and present findings of interest to the HF community. Addressing the statistical, methodological, and interpretive concerns outlined above would substantially enhance the validity of the manuscript's conclusions. We look forward to prospective studies that more definitively establish the prognostic role of PR severity in HF populations.
