# Supplementary material for: Insuficiência Pulmonar na Insuficiência Cardíaca: Causalidade ou Marcador de Gravidade da Doença?
Source: Arq Bras Cardiol. 2026 Jul 30;123(7):e20260265. [Article in Portuguese] doi: 10.36660/abc.20260265 (PMC13668446; doi:10.36660/abc.20260265)
Supplement: Related texts

# Reply: Carta-resposta

**Authors:** Fatih Aydin, Bektas Murat, Selda Murat, Muhammet Burak Daghan

**Affiliations:** 1 TC Sağlik Bakanliği Eskişehir Şehir Hastanesi Odunpazari Turquia TC Sağlik Bakanliği Eskişehir Şehir Hastanesi, Odunpazari – Turquia; 2 Eskisehir City Hospital Eskisehir Turquia Eskisehir City Hospital, Eskisehir – Turquia; 3 Eskisehir Osmangazi University Medical Faculty Department of Cardiology Eskisehir Turquia Eskisehir Osmangazi University – Medical Faculty Department of Cardiology, Eskisehir – Turquia

**Prezado Editor,**

Agradecemos aos autores da carta pela leitura atenta do nosso trabalho e por levantarem diversos pontos que nos permitem esclarecer o desenho e a interpretação do nosso estudo. Acolhemos com satisfação este tipo de diálogo acadêmico e esperamos que nossa resposta aborde cada uma das questões levantadas.

Em relação ao desequilíbrio no tamanho dos grupos e ao poder estatístico, reconhecemos plenamente a distribuição desigual entre as categorias de gravidade da RP. Isso reflete a prevalência real da regurgitação pulmonar em uma população geral com insuficiência cardíaca, onde as formas moderada e grave são inerentemente menos comuns. O objetivo do nosso estudo foi justamente caracterizar essa distribuição como ela existe na prática clínica, e não equilibrar os grupos artificialmente. Dito isso, a questão referente aos intervalos de confiança para os coeficientes de regressão é pertinente. Reconhecemos que apresentar os intervalos de confiança de 95% juntamente com os coeficientes beta padronizados aumentaria a transparência. Análises de sensibilidade por *bootstrap* são uma sugestão razoável para iterações futuras, e acolhemos essa orientação para trabalhos prospectivos.

Em relação à confiabilidade da classificação ecocardiográfica, os comentaristas observam corretamente a ausência de estatísticas kappa formais. Devemos enfatizar, no entanto, que nosso protocolo de classificação não se baseou apenas na largura da vena contracta. Conforme descrito na seção Métodos, empregamos uma abordagem abrangente e multiparamétrica que integrou a largura do jato Doppler em cores em relação ao anel pulmonar, a densidade do sinal Doppler de onda contínua e o perfil de desaceleração, além da presença de fluxo diastólico reverso nos ramos pulmonares, o que está totalmente de acordo com as recomendações da Sociedade Americana de Ecocardiografia.^1^ A largura da vena contracta foi destacada por ser o único parâmetro disponível retrospectivamente em

formato tabular; ela não abrange toda a extensão da nossa abordagem de classificação. Concordamos que a análise formal de concordância interobservador deve ser um pilar fundamental de qualquer estudo prospectivo futuro.

A preocupação com o viés de seleção no TC6M é legítima e foi explicitamente discutida na seção de Limitações. No entanto, gostaríamos de questionar essa abordagem. Se os pacientes com a limitação funcional mais grave de fato não conseguissem completar o TC6M, as diferenças observadas entre os grupos de gravidade da doença de Parkinson representariam uma estimativa conservadora do verdadeiro gradiente funcional, e não uma superestimação. Isso, se algo, tornaria nossos achados mais, e não menos, confiáveis. Mesmo assim, concordamos que relatar as taxas de conclusão por grupo é uma medida valiosa de transparência, e futuros estudos prospectivos devem incorporar essa informação.

Finalmente, aceitamos integralmente a crítica referente à linguagem causal e aos fatores de confusão não mensurados. A carga de fibrilação atrial, o índice de massa corporal, a etiologia da IC e o uso de inibidores de SGLT2 ou sacubitril/valsartana são variáveis relevantes que nossos dados retrospectivos não capturaram de forma consistente. A via causal inversa, na qual a IC avançada leva à hipertensão pulmonar secundária, dilatação do anel e, por fim, à RP, é uma explicação alternativa biologicamente plausível e importante.^2,3^ Não tínhamos a intenção de sugerir um mecanismo unidirecional e concordamos que a linguagem mecanicista deve ser consistentemente substituída por uma abordagem associativa. Nossas conclusões são explicitamente observacionais e quaisquer implicações terapêuticas devem ser interpretadas como geradoras de hipóteses, e não como orientações práticas.^4,5^

Agradecemos aos correspondentes por contribuírem para o aprimoramento da discussão sobre essa lesão valvar pouco explorada no contexto da insuficiência cardíaca. Seus comentários reforçam a necessidade de estudos de coorte prospectivos bem delineados, os quais pretendemos realizar.

## References

[1] Zoghbi WA, Adams D, Bonow RO, Enriquez-Sarano M, Foster E, Grayburn PA (2017). Recommendations for Noninvasive Evaluation of Native Valvular Regurgitation: A Report from the American Society of Echocardiography Developed in Collaboration with the Society for Cardiovascular Magnetic Resonance. J Am Soc Echocardiogr.

[2] Lancellotti P, Tribouilloy C, Hagendorff A, Popescu BA, Edvardsen T, Pierard LA (2013). Recommendations for the Echocardiographic Assessment of Native Valvular Regurgitation: An Executive Summary from the European Association of Cardiovascular Imaging. Eur Heart J Cardiovasc Imaging.

[3] Thandavarayan RA, Chitturi KR, Guha A (2020). Pathophysiology of Acute and Chronic Right Heart Failure. Cardiol Clin.

[4] McDonagh TA, Metra M, Adamo M, Gardner RS, Baumbach A, Böhm M (2021). 2021 ESC Guidelines for the Diagnosis and Treatment of Acute and Chronic Heart Failure. Eur Heart J.

[5] Grimes DA, Schulz KF (2002). Bias and Causal Associations in Observational Research. Lancet.

# Reply

**Authors:** Fatih Aydin, Bektas Murat, Selda Murat, Muhammet Burak Daghan

**Affiliations:** 1 TC Sağlik Bakanliği Eskişehir Şehir Hastanesi Odunpazari Türkiye TC Sağlik Bakanliği Eskişehir Şehir Hastanesi, Odunpazari – Türkiye; 2 Eskisehir City Hospital Eskisehir Türkiye Eskisehir City Hospital, Eskisehir – Türkiye; 3 Eskisehir Osmangazi University – Medical Faculty Department of Cardiology Eskisehir Türkiye Eskisehir Osmangazi University – Medical Faculty Department of Cardiology, Eskisehir – Türkiye

**Dear Editor,**

We thank the authors of the letter for their careful reading of our work and for raising several points that allow us to clarify the design and interpretation of our study. We welcome this kind of scholarly dialogue, and we hope our response addresses each concern in turn.

Regarding group size imbalance and statistical power, we fully acknowledge the unequal distribution across PR severity categories. This reflects the real-world prevalence of pulmonary regurgitation in a general heart failure population, where moderate and severe forms are inherently less common. The purpose of our study was precisely to characterize this distribution as it exists in clinical practice, not to balance groups artificially. That said, the point concerning confidence intervals for the regression coefficients is well taken. We concede that reporting 95% confidence intervals alongside the standardized beta coefficients would strengthen transparency. Bootstrap sensitivity analyses are a reasonable suggestion for future iterations, and we welcome this guidance for prospective work.

On echocardiographic grading reliability, the commentators correctly note the absence of formal kappa statistics. We must emphasize, however, that our grading protocol was not based solely on vena contracta width. As described in the Methods section, we employed a comprehensive, multi-parameter approach that integrated color Doppler jet width relative to the pulmonary annulus, continuous-wave Doppler signal density and deceleration profile, and the presence of diastolic flow reversal in the pulmonary branches, which is fully consistent with the American Society of Echocardiography recommendations.^1^ Vena contracta width was highlighted because it was the only parameter available in tabular form retrospectively; it does not capture the full scope of our classification approach. We agree that formal interobserver analysis should be a cornerstone of any future prospective study.

The concern about ascertainment bias in the 6MWT is legitimate and is one we explicitly discussed in the Limitations section. However, we would gently push back on the framing. If patients with the most severe functional limitation were indeed unable to complete the 6MWT, the observed differences across PR severity groups would represent a conservative estimate of the true functional gradient rather than an overestimation. This would, if anything, make our findings more rather than less credible. Nevertheless, we agree that reporting completion rates per group is a valuable transparency measure, and future prospective designs should incorporate this.

Finally, we fully accept the critique regarding causal language and unmeasured confounders. Atrial fibrillation burden, body mass index, HF etiology, and the use of SGLT2 inhibitors or sacubitril/valsartan are relevant variables that our retrospective data did not consistently capture. The reverse causal pathway, in which advanced HF drives secondary pulmonary hypertension, annular dilation, and ultimately PR, is a biologically plausible and important alternative explanation.^2,3^ We did not intend to imply a unidirectional mechanism, and we agree that mechanistic language should consistently be replaced with associative framing. Our conclusions are explicitly observational, and any therapeutic implications should be read as hypothesis-generating rather than actionable guidance.^4,5^

We are grateful to the correspondents for helping to sharpen the discussion of this underexplored valvular lesion in the context of heart failure. Their comments reinforce the need for well-designed prospective cohort studies, which we intend to pursue.
